# Effects of Acupuncture Combined with Moxibustion on Reproductive and Metabolic Outcomes in Patients with Polycystic Ovary Syndrome: A Systematic Review and Meta-Analysis

**DOI:** 10.1155/2022/3616036

**Published:** 2022-03-31

**Authors:** Peishuang Li, Jiahua Peng, Zhiling Ding, Xu Zhou, Ruining Liang

**Affiliations:** ^1^Institute of Obstetrics and Gynecology, Jiangxi University of Chinese Medicine, Jiangxi, Nanchang 330006, China; ^2^Evidence-based Medicine Research Center, Jiangxi University of Chinese Medicine, Jiangxi, Nanchang 330006, China

## Abstract

**Objectives:**

In this systematic review, the effects of acupuncture combined with moxibustion on reproductive and metabolic outcomes in patients with polycystic ovary syndrome (PCOS) were evaluated.

**Methods:**

Randomized controlled trials (RCTs) assessing acupuncture combined with moxibustion + basic treatment (experimental group) versus basic treatment alone (control group) for treating PCOS were identified from English and Chinese databases up to November 3, 2021. Outcomes related to pregnancy, ovulation, miscarriage, sex hormones, and metabolic disorders were of interest. In the meta-analysis, risk ratios (RRs) and mean differences (MDs) and their 95% confidence intervals (CIs) were used as effect measures.

**Results:**

Twenty-five RCTs (*n* = 1991) were included. The pooled results showed that the experimental group had significant increases in the pregnancy rate (RR 1.81, 95% CI 1.58 to 2.08) and ovulation rate (RR 1.31, 95% CI 1.22 to 1.40) and decreases in the miscarriage rate (RR 0.45, 95% CI 0.28 to 0.73), and ovarian volume (MD −0.75 cm^3^, 95% CI −1.30 to −0.20). In the experimental group, improvements in the luteinizing hormone (LH) level, the LH-to-follicle-stimulating hormone (FSH) ratio, total testosterone level, fasting insulin level, and body mass index, but not in FSH, oestradiol, or dehydroepiandrosterone sulfate levels, were significantly greater. All reported adverse events were mild. Based on the limitations of risk of bias, inconsistency, imprecision, and/or publication bias, the level of evidence was judged to be moderate for the pregnancy rate, ovulation rate, miscarriage rate, LH level, and LH/FSH ratio and very low for the other outcomes.

**Conclusion:**

Among patients with PCOS, using acupuncture combined with moxibustion as a complementary therapy to basic treatments can improve pregnancy, ovulation, and miscarriage rates, the levels some sex hormones, and metabolic indicators, with good safety. Additionally, this combination therapy may have no effect on the FSH, oestradiol, or dehydroepiandrosterone sulfate level.

## 1. Introduction

Polycystic ovary syndrome (PCOS), an endocrinopathy, is the most common cause of reproductive impairment in women of reproductive age. In their consensus conference in 2003, the European Society of Human Reproduction and Embryology (ESHRE) and the American Society for Reproductive Medicine (ASRM) defined the PCOS as meeting at least two of the following three criteria, namely, hyperandrogenism, hypo-ovulation/anovulation, and polycystic ovaries, and these are currently the most universally accepted diagnostic criteria [1]. Globally, in 2017, the age-standardized incidence of PCOS globally was 82.4 per 100,000, representing a 1.45% increase compared to 10 years prior [2], and the total prevalence of PCOS was estimated to be 5–15% [3]. The predominant negative effect of PCOS is that it causes reproductive disorders; 70–80% of patients with PCOS develop anovulatory infertility [4], and patients with PCOS are also more likely to experience miscarriages (16 versus 5.3% among healthy controls) [5]. PCOS also causes amenorrhea, acne, and hirsutism and is associated with metabolic diseases related to insulin resistance (e.g., diabetes and central obesity) [6, 7].

PCOS is not curable to date, and there are no specific drugs approved for PCOS. Therefore, clinical treatment for PCOS is symptom-oriented. For anovulatory infertility, treatment options include drugs, surgery, and in vitro fertilization. Unfortunately, these treatments are limited by uncertain efficacy, side-effect concerns, and high costs [6]. A 6-month course of clomiphene, a first-line drug for ovulation induction, is associated with a pregnancy rate of only 67% and may cause side effects such as flushing, headache, visual disturbances, and abdominal discomfort [8]. The natural pregnancy rate after ovarian drilling is 54–70%, whereas surgery is invasive and may cause premature ovarian insufficiency [9]. In vitro fertilization is expensive, and patients undergoing this treatment carry a risk of ovarian hyperstimulation syndrome [10]. In addition, the long-term use of cyproterone, a commonly used androgen receptor blocker against hyperandrogenemia, may even cause fatal pulmonary embolism [11]. Therefore, there is a need to seek more complementary and alternative therapies for the treatment of PCOS, particularly with regard to both efficacy and safety.

In China, acupuncture is prevalent as a complementary and alternative therapy. It is performed by inserting filiform needles into the skin to stimulate specific acupoints; additionally, pairs of needles can be connected with continuous electrical pulses to enhance stimulation, a procedure known as electroacupuncture. Acupuncture has been used to treat endocrine and metabolic diseases such as obesity and diabetes (and its consequent neuropathy), and its efficacy for treating these diseases is established [12–14]. Notably, acupuncture is widely used to treat PCOS, but a recent large factorial trial (the PCOS Acupuncture and Clomiphene Trial, PCOSAct) negated both the reproductive and metabolic benefits of acupuncture for patients with PCOS [15]. The study generated considerable controversy after publication, especially because many acupuncturists believed that the reason why acupuncture was ineffective was that the regimen was not optimal [16, 17].

For instance, in clinical practice, specifically in the acupuncture and moxibustion departments of Chinese hospitals, acupuncture is often administered in combination with moxibustion to achieve better efficacy. Moxibustion is also an acupoint stimulation therapy that is performed by igniting a moxa stick and placing it on the acupoints to deliver thermal stimulation. There are two strategies for combining acupuncture and moxibustion: (1) warm-needle acupuncture, namely, attaching a burning moxa cone to the needle after insertion; and (2) the separate application of acupuncture and moxibustion, wherein different forms of moxibustion, such as suspended moxibustion, herb-separated moxibustion, and heat-sensitive moxibustion, can be performed. The combined use of acupuncture and moxibustion has been shown to have advantages over acupuncture alone in treating multiple diseases, such as knee osteoarthritis [18], low back pain [19], and stroke management [20].

To date, there is no lack of animal experiments or systematic review evidence on the efficacy of acupuncture or moxibustion for PCOS [21–23]. A number of randomized controlled trials (RCTs) of acupuncture combined with moxibustion for treating PCOS in humans have also been published, but their conclusions have been inconsistent. Considering that these RCTs were predominantly small studies, some of the negative results may be due to type 2 error caused by an insufficient sample size. Therefore, we conducted a systematic review of currently available RCTs to draw more precise conclusions via meta-analysis techniques to inform clinical practice regarding the use of acupuncture combined with moxibustion to treat PCOS.

## 2. Materials and Methods

This systematic review was developed with the guidance of the Preferred Reporting Items for Systematic Reviews and Meta-Analyses statement [24], and its protocol was prospectively registered in the PROSPERO database with the number CRD42021284400.

### 2.1. Literature Search

We searched seven literature databases (PubMed, EMBASE, Cochrane Controlled Register of Trials, CNKI, Wanfang, VIP, and Chinese Biomedical Literature), two preprint platforms (MedRxiv and bioRxiv), and two clinical trial registration databases (Clinicaltrials.gov and Chinese Clinical Trial Registry) to identify RCTs published from database inception to November 3, 2021. Keywords related to PCOS, acupuncture, and moxibustion were used, without limitations on language or publication status (see details in Table S1 in Supplementary Materials). Relevant narrative reviews and systematic reviews were examined to identify additional studies.

### 2.2. Eligibility Criteria

Parallel-group RCTs were included if they investigated the association of the combined use of acupuncture and moxibustion with PCOS. The diagnosis of PCOS could be based on any criteria consistent with the ESHRE/ASRM 2003 consensus [1]. Eligible interventions were manual acupuncture or electroacupuncture combined with moxibustion, regardless of how these two interventions were performed. Studies of thermotherapy not involving moxa materials, such as infrared laser moxibustion, were excluded. The comparison had to be between (1) acupuncture and moxibustion + medications for PCOS and the same medications; or (2) acupuncture and moxibustion and lifestyle/no intervention. Acupuncture and moxibustion were not allowed in the control group. Studies involving other acupoint stimulation therapies in either group, such as acupressure, cupping, and acupoint catgut embedding, were excluded. Conference abstracts, repeated reports, and literature lacking the complete data required for meta-analysis were also excluded.

### 2.3. Outcomes

The primary outcomes were defined as the pregnancy rate after the treatments, counting both biochemical and clinical pregnancies and using the number of patients with a need for pregnancy as the denominator. The secondary outcomes included ovulation rate, miscarriage rate, change in ovarian volume, change in hormones (luteinizing hormone [LH] level, follicle-stimulating hormone [FSH] level, LH/FSH ratio, total testosterone level, oestradiol level, and dehydroepiandrosterone sulfate [DHEAS] level), change in fasting insulin level, change in body mass index (BMI), and incidence of adverse events.

### 2.4. Literature Screening and Data Extraction

Literature screening and data extraction were independently performed by two reviewers. They first checked titles and abstracts to discard irrelevant records and then read the full texts of the remaining studies to determine which would be included. Baseline characteristics (sample size, sex proportion, age, and duration of disease), treatment details (type, dose, and course), and outcome data were extracted from each included RCT. If there were outcome data for multiple time points, we retained only the data from the last follow-up visit. For crossover trials, we utilized the data only before its wash-out period. A third reviewer resolved disagreements during these processes.

### 2.5. Risk-of-Bias Assessment

We used a modified version of the Cochrane risk-of-bias assessment tool for RCTs [25]. The domains assessed were the same as the original scale, including random sequence generation, allocation concealment, blinding of patients and physicians, blinding of assessors and analysts, data completeness, selective reporting, and other potential for risk of bias. In addition to making “yes (low risk)” and “no (high risk)” judgments for each domain, the tool also requires a reasonable inference of “probably yes” or “probably no” for “unclear” items based on relevant information in the reporting. The overall risk of bias in the included RCTs was graded as low, moderate, or high based on the following criteria: (1) overall low risk indicated that all items were judged as yes or probably yes, in which the unblinding of patients and physicians was allowed because it is impossible in this topic; (2) overall high risk indicated that more than three items were judged as no or probably no; and (3) overall moderate risk included the cases that did not fit the above two sets of criteria. The assessment was independently performed by two reviewers, and disagreements were resolved by a third reviewer.

### 2.6. Statistical Analysis

We performed random-effects meta-analyses to incorporate data from individual RCTs. Effects on binary outcomes were measured by means of risk ratios (RRs) and pooled by the Mantel–Haenszel method, in which 0.5 was added to the zero events. Effects on continuous outcomes were measured by mean differences (MDs) and pooled by the inverse variance method, in which missing standard deviations in the change from baseline assessment were imputed by the Cochrane handbook method with a correlation coefficient of 0.5. Confidence intervals (CIs) and prediction intervals were also calculated to assess the imprecision of effect estimates.

The heterogeneity between summary data was tested using Cochran's *Q* test and the *I*^2^ statistic. A *P* value in the *Q* test of less than 0.10 or an *I*^2^ of greater than 50% represented statistically significant heterogeneity [26]. To identify the source of heterogeneity, we planned the following subgroup analyses with an anticipated effect direction [27]: (1) type of cointervention: Western medicine + Chinese herbal medicine (anticipated to be the best option) versus Western medicine versus Chinese herbal medicine versus lifestyle intervention; (2) type of acupuncture: electroacupuncture (anticipated to be the better option) versus manual acupuncture; (3) type of moxibustion: warm-needle moxibustion (anticipated to be the better option) versus other forms; and (4) course of treatment: ≥3 months (anticipated to be the better option) versus <3 months.

Sensitivity analyses were performed to test the robustness of the estimates, including the exclusion of studies with a high overall risk of bias, studies that counted the number of menstrual cycles rather than the number of patients in the analysis of ovulation rate, and studies using a fixed-effect model for meta-analyses with nonsignificant heterogeneity. If there were 10 or more studies included in a meta-analysis, we detected publication bias by drawing funnel plots and conducting the Thompson test (for binary outcomes) or Egger's test (for continuous outcomes). The “meta” package in *R* version 4.0.2 (Ross Ihaka, Robert Gentlemen, New Zealand) was used for the statistics and plotting.

Finally, we evaluated the level of evidence for each outcome using the Grading of Recommendations, Assessment, Development, and Evaluations (GRADE) approach and the Confidence in Network Meta-Analysis (CINeMA) guidelines [28]. As the meta-analyses were conducted based on RCTs, the initial level of evidence was high. If the meta-analytic results suffered from within-study bias, inconsistency, imprecision, indirectness, or publication bias, the level of evidence was downgraded to moderate, low, or very low according to the guidelines.

## 3. Results

### 3.1. Study Screening

**Figure 1:**
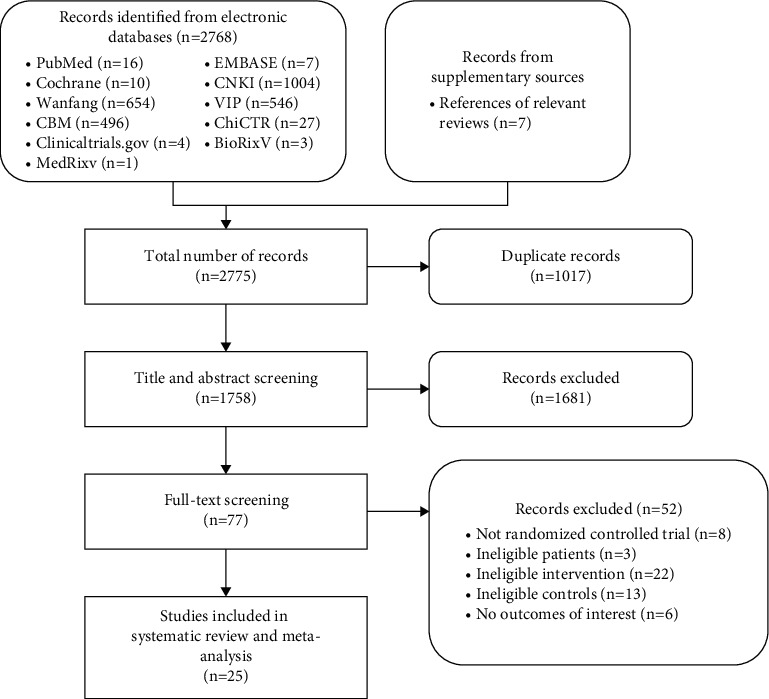
Flowchart of study screening.

The search yielded 2768 records, and 25 RCTs [29–53] were ultimately included in the systematic review after screening (Figure 1). One of the RCTs [31] had two independent pairs of comparisons, which we treated as two studies in the data analysis.

### 3.2. Characteristics of the Included Studies

The basic characteristics of the included RCTs are compiled in Table 1. In summary, a total of 1991 patients were enrolled in the RCTs, with a sample size ranging from 60 to 200 and a mean age of 25.3 to 34.5 years. The mean course of PCOS ranged from 1.43 to 4.94 years. Electroacupuncture was applied in 5 trials, and the remaining 21 trials used only manual acupuncture. The type of moxibustion was warm-needle moxibustion in 16 trials, suspended moxibustion in five trials, moxibustion box in three trials, and ginger-separated and thunder-fire moxibustion in one trial each. The cointervention in both groups was Western medicine in 16 trials, Chinese herbal medicine in 6 trials, Western medicine + Chinese herbal medicine in 2 trials, and lifestyle intervention in 1 trial. The course of treatment was ≤3 months in 17 trials >3 months in five trials and unclear in the rest. The mean baseline LH and total testosterone levels were higher than the normal range in four [32, 41, 42, 48] and seven trials [32, 34, 39, 41, 42, 46, 51], respectively, and the mean baseline FSH level was normal in all trials. Detailed information regarding the acupoint selection and Chinese medicine formulas can be found in Table S2 and Table S3 in the Supplementary Materials.

### 3.3. Results of the Risk-of-Bias Assessment

**Figure 2:**
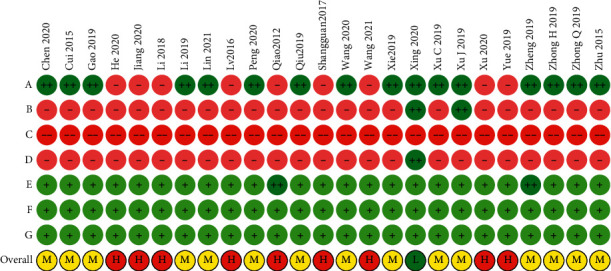
Risk-of-bias assessment (a) random number generation; (b) allocation concealment; (c) blinding of patients and clinicians; (d) blinding of assessors and analysts; (e) data completeness; (f) selective reporting; (g) other sources of bias; “++”: low risk; “+”: probably low risk; “−”: probably high risk; (h) overall high risk; (m) overall moderate risk; (l); overall low risk.

Sixteen trials used a correct method to generate random numbers (12 used a random number table [29, 31, 35, 36, 40, 42, 44, 47, 50–53], three used computer software [30, 45, 46], and one used a lottery method [38]). Sealed envelopes [47] and central randomization [45] were used in one trial each to conceal the allocation. The assessors and analysts were blinded in one trial [45]. Two trials reported zero attrition [40, 50], and five trials reported an attrition rate of less than 10% [30, 36, 37, 42, 46]. The remaining trials did not report the attrition rate, but we inferred that they probably completed the follow-up for all patients. All the trials were probably free from selective reporting and other biases. Overall, only one trial was considered to have a low risk of bias [45], 15 had a moderate risk of bias [29–31, 35, 36, 38, 40, 42, 44, 46, 47, 50–53], and nine had a high risk of bias [32–34, 37, 39, 41, 43, 48, 49] (Figure 2).

### 3.4. Outcomes of Pregnancy and Ovulation

#### 3.4.1. Pregnancy Rate

**Figure 3:**
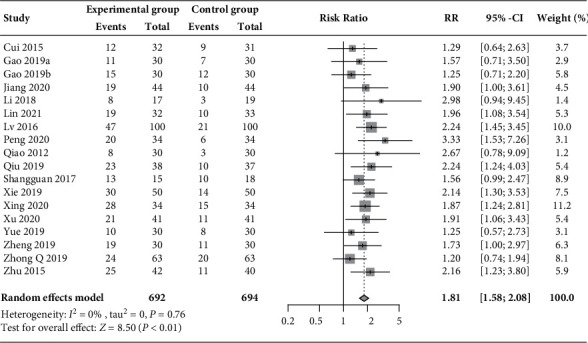
Forest plot of meta-analysis of pregnancy rate.

With regard to the pregnancy rate, pooled results from 18 RCTs involving 1386 patients with PCOS [30, 31, 33, 34, 36–41, 44, 45, 48–50, 52, 53] showed that an increase in the pregnancy rate was associated with the additional use of acupuncture and moxibustion compared with medication treatment alone (50.9% vs. 27.5%; RR 1.81, 95% CI 1.58 to 2.08, *P* < 0.01; Figure 3), without statistical heterogeneity (*I*^2^ = 0%).

#### 3.4.2. Ovulation Rate

**Figure 4:**
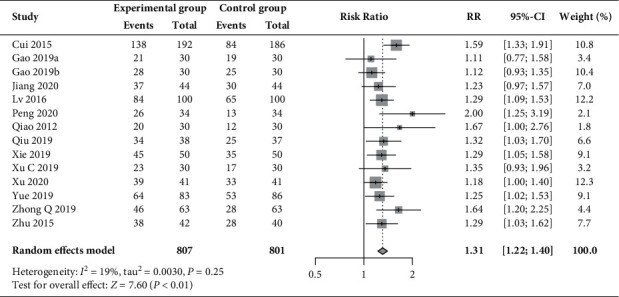
Forest plot of the meta-analysis of ovulation rate.

As shown in Figure 4, a significant increase in the ovulation rate with low heterogeneity (*I*^2^ = 19%) was also found in the pooled result of 14 RCTs [30, 31, 33, 37–40, 44, 46, 48, 49, 52, 53] that compared the additional use of acupuncture plus moxibustion with medication alone (79.7% vs. 58.3%; RR 1.31, 95% CI 1.22 to 1.40, *P* < 0.01).

#### 3.4.3. Miscarriage Rate

**Figure 5:**
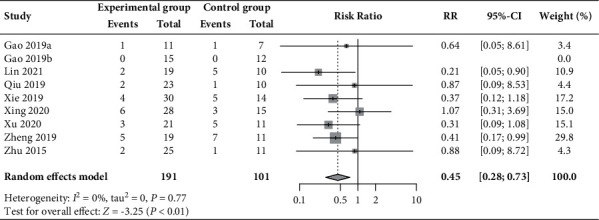
Forest plot of the meta-analysis of miscarriage rate.

In all, 13.1% (25/191) and 27.7% (28/101) of patients experienced miscarriage in the experimental and control groups, respectively, as reported in nine RCTs [31, 36, 40, 44, 45, 48, 50, 53]. The between-group difference was statistically significant (RR 0.45, 95% CI 0.28 to 0.73, *P* < 0.01; Figure 5), without heterogeneity (*I*^2^ = 0%).

#### 3.4.4. Ovarian Volume

**Figure 6:**
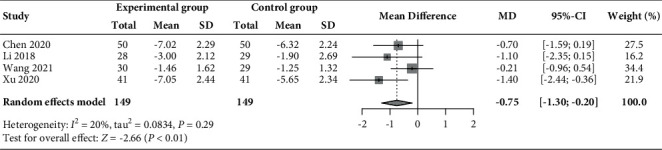
Forest plot of meta-analysis of changes in ovarian volume (cm^3^).

Four RCTs [29, 34, 43, 48] reported data on changes in ovarian volume before and after treatment. The pooled results (Figure 6) showed that a significant reduction in ovarian volume was associated with the additional use of acupuncture and moxibustion compared with the basic treatment alone (MD −0.75 cm^3^, 95% CI −1.30 to −0.20, *P* < 0.01; *I*^2^ = 20.4%).

### 3.5. Sex Hormones

#### 3.5.1. LH and FSH

**Figure 7:**
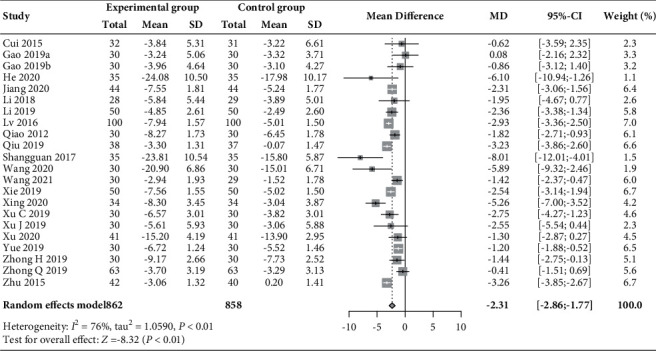
Forest plot of meta-analysis of changes in LH (mIU/mL).

**Figure 8:**
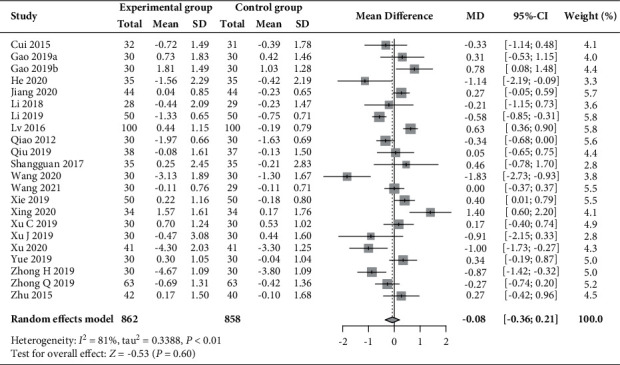
Forest plot of meta-analysis of changes in FSH (mIU/mL).

**Figure 9:**
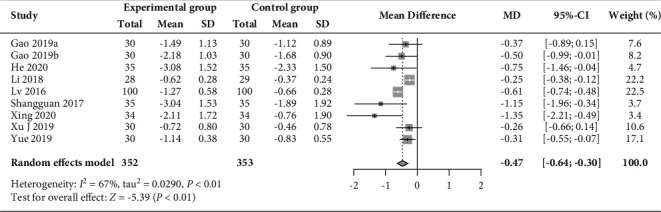
Forest plot of meta-analysis of changes in the LH/FSH ratio.

Twenty-two RCTs (*n* = 1720) [30–35, 37, 39–49, 51–53] reported on changes in the levels of LH and FSH, and 9 of these RCTs (*n* = 705) [31, 32, 34, 37, 41, 45, 47, 49] had data on the LH/FSH ratio. As shown in Figures 7–9, compared to those receiving basic treatment alone, patients receiving acupuncture combined with moxibustion had a significantly reduced LH level (MD –2.31 mIU/mL, 95% CI −2.86 to −1.77, *P* < 0.01; *I*^2^ = 76%) and LH/FSH ratio (MD −0.47, 95% CI −0.64 to −0.30, *P* < 0.01; *I*^2^ = 67%). However, the combined use of acupuncture and moxibustion did not significantly impact the level of FSH (MD −0.08 mIU/mL, 95% CI −0.36 to 0.21, *P*=0.60; *I*^2^ = 81%).

#### 3.5.2. Total Testosterone and DHEAS

**Figure 10:**
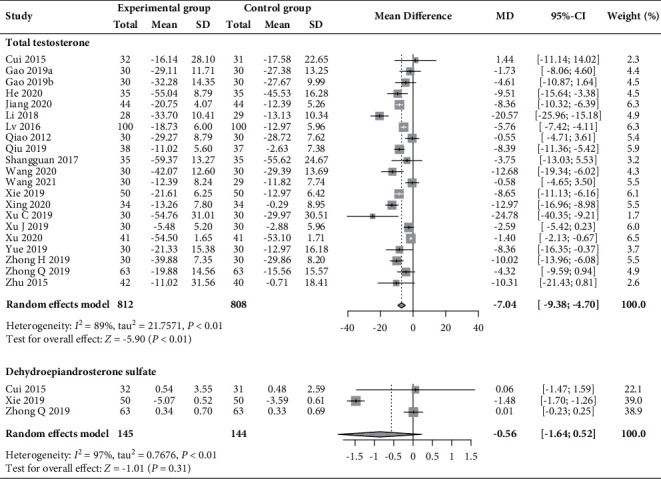
Forest plot of meta-analysis of changes in total testosterone (ng/dL) and dehydroepiandrosterone sulfate (*μ*mol/L).

Data pooled from 21 RCTs (*n* = 1620) [30–34, 37, 39–49, 51–53] revealed that compared to basic treatment alone, acupuncture plus moxibustion significantly reduced the level of total testosterone (MD −7.04 ng/dL, 95% CI −9.38 to −4.70, *I*^2^ = 89%; Figure 9). However, pooled data from three RCTs (*n* = 145) [30, 44, 52] indicated no clear benefit of acupuncture plus moxibustion on the DHEAS level (MD −0.56 *μ*mol/L, 95% CI −1.64 to 0.52; *I*^2^ = 97%; Figure 10).

#### 3.5.3. Oestradiol

**Figure 11:**
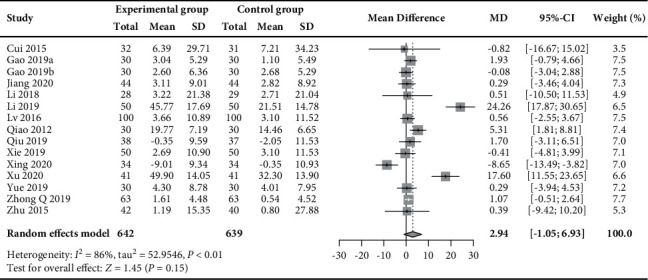
Forest plot of meta-analysis of changes in oestradiol (pg/mL).

Pooling of data for oestradiol was possible for 15 RCTs (*n* = 1281) [30, 31, 33–35, 37, 39, 40, 44, 45, 48, 49, 52, 53] and showed that there was no significant change in oestradiol when acupuncture plus moxibustion was administered (MD 2.94 pg/mL, 95% CI −1.05 to 6.93, *I*^2^ = 86%; Figure 11).

### 3.6. Outcomes of Metabolism

**Figure 12:**
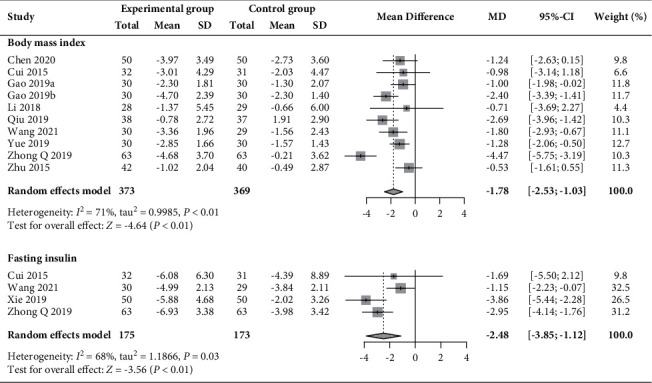
Forest plot of meta-analysis of changes in BMI (kg/m^2^) and fasting insulin (mIU/L).

Ten [29–31, 34, 40, 43, 49, 52, 53] and four trials [30, 43, 44, 52] contributed data regarding BMI and fasting insulin level, involving 742 and 348 patients, respectively. As shown in Figure 12, the experimental group had greater reductions in both BMI (MD −1.78 kg/m^2^, 95% CI −2.53 to −1.03, *I*^2^ = 71%) and fasting insulin level (MD −2.48 mIU/L, 95% CI −3.85 to 1.12, *P* < 0.01; *I*^2^ = 68%) than the control group.

### 3.7. Additional Analysis

#### 3.7.1. Subgroup Analysis

A significant subgroup difference (indicated by an interaction *P* value < 0.05) was found in the subgroup analyses stratified by the basic treatments for the ovulation rate (Western medicine was the best, interaction *P*=0.01) and for the level of FSH (Western medicine combined with Chinese herbal medicine was the best, *P*=0.01); in the analyses stratified by the basic treatments (Chinese herbal medicine was the best, *P*=0.01), type of acupuncture (manual acupuncture was better, *P*=0.01), and type of moxibustion (nonwarm-needle moxibustion was better, *P*=0.03) for the LH level; in the analyses stratified by the basic treatments (Western medicine was the best, *P* < 0.01), type of acupuncture (manual acupuncture was better, *P* < 0.01), type of moxibustion (warm-needle moxibustion was better, *P* < 0.01), and course of treatment (≤3 months was better, *P*=0.04) for the LH/FSH ratio; and in the analyses stratified by the course of treatment (>3 months was better, *P* < 0.01) for the total testosterone level. Details of the subgroup analyses are shown in Table S4 in the Supplementary Materials.

#### 3.7.2. Sensitivity Analysis

A change in the effect direction was found for the ovarian volume after excluding studies with a high risk of bias (MD −0.70 [95% CI −1.59 to 0.19] vs. main analysis: MD −0.75 [95% CI −1.30 to −0.20]). No other important changes were found. Details of the sensitivity analyses are presented in Table S5 in the Supplementary Materials.

#### 3.7.3. Publication Bias

**Figure 13:**
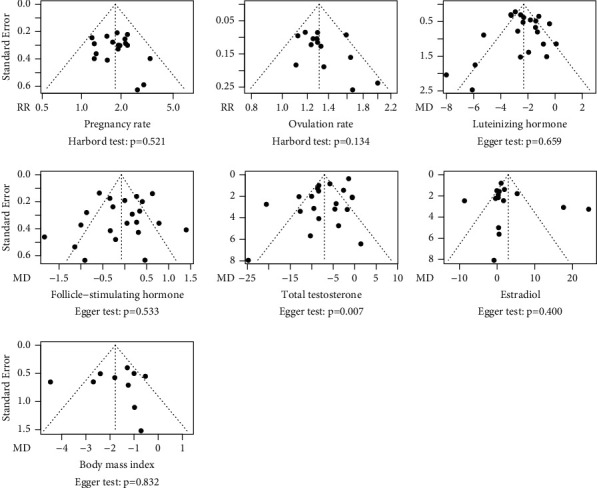
Funnel plot and results of Harbord's or Egger's tests. Outcomes with an insufficient number of studies to detect publication bias are not presented.

As shown in Figure 13, a significant publication bias was found for only the analysis of the total testosterone level, indicated by an asymmetric funnel plot and a *P* value of 0.007 by Egger's test.

### 3.8. Level of Evidence

The meta-analytic results for the pregnancy rate, ovulation rate, miscarriage rate, and LH level were associated with a serious risk of bias and were downgraded to a moderate level of evidence. The levels of evidence of all remaining outcomes were judged to be very low because of serious or very serious limitations regarding risk of bias, inconsistency, imprecision, and/or publication bias. The GRADE evidence profiles are compiled in Table S6 in the Supplementary Materials.

### 3.9. Safety

Seven trials [31, 34, 39, 45, 46, 49, 50] reported safety data. There were two cases of subcutaneous bleeding and two cases of pain at the acupuncture site in Xing et al.'s study [45], one case of pelvic pain in Xu's study [46], and two cases of needle sickness in Yue et al.'s study [49]. These events were mild, and remission was achieved without specific treatment. The remaining trials claimed that no adverse events occurred.

## 4. Discussion

The systematic review included 25 RCTs assessing the efficacy and safety of acupuncture combined with moxibustion in treating PCOS, and all of them contributed data to the meta-analysis. Evidence of an association between acupuncture and moxibustion therapy and greater increases in the pregnancy rate and ovulation rate and greater reductions in the miscarriage rate and ovarian volume was found. Additionally, patients receiving acupuncture and moxibustion also exhibited greater improvements in some sex hormones (LH level, LH/FSH ratio and total testosterone level) and indicators related to metabolic disorders (fasting insulin level and BMI). Nevertheless, acupoint stimulation therapy had no significant effect on the levels of FSH, DHEAS, or oestradiol.

In this systematic review, the pregnancy rate among patients who received acupuncture combined with moxibustion reached 50.9%. This value represents a substantial increase compared to that of patients who did not receive acupuncture (27.5%), with an RR of 1.81 and a 4.9% additional increase compared with patients receiving acupuncture + active drugs in the PCOSAct trial (46.0%) [15]. In terms of the miscarriage rate, the additional use of moxibustion had an even greater advantage (the experimental group in this review vs. acupuncture + active drugs group in the PCOSAct trial: 13.1% vs. 35.2% [15]). Although such cross-study comparisons may not be precise, we think that moxibustion probably has a synergistic effect on acupuncture in the treatment of PCOS; notably, this synergistic effect has been observed in the treatment of other disorders [54–56].

One of the main pathophysiological states of PCOS is the disturbed hypothalamic–pituitary–ovarian/adrenal axis. This state results in increased LH release, a decreased FSH level, and a reversed increase in the LH/FSH ratio, leading to cessation of ovulation and infertility [57]. Our analysis showed that both the LH level and the LH/FSH ratio were significantly reduced after acupuncture and moxibustion treatment but that improvements were not found in the levels of FSH or oestradiol. Therefore, the role of acupuncture combined with moxibustion in lowering the LH/FSH ratio should be attributed to its inhibitory effect on LH. Mechanistically, excessive LH secretion in PCOS is related to increased pituitary sensitivity to gonadotropin-releasing hormone and changes in its secretion pattern [58]. Stimulation at the acupoint has been shown to raise the level of *β*-endorphin in the central endocrine system and peripheral circulation, which is involved in the direct and indirect tonic inhibition of gonadotropin-releasing hormone and subsequent LH release [59]. Moreover, altered sympathetic neurogenic control of the ovary is associated with the pathogenesis of PCOS [56], and stimulation at the acupoint can also reduce sympathetic activity by inhibiting the overexpression of nerve growth factor, which may result in a return to normal levels of the ovarian steroid response to gonadotropins [60].

Most patients with PCOS suffer from chronically elevated levels of androgens in the ovaries and circulatory system. Hyperandrogenemia can enhance the activity of mitogen-activated protein kinase in endometrial cells by activating mitogen-activated protein kinase, subsequently inducing endometrial hyperplasia [61]. It can also reduce endometrial tolerance by disrupting the expression of homeobox A10, which controls endometrial differentiation and is closely associated with infertility and recurrent abortion [62]. We found that acupuncture and moxibustion significantly reduced the total testosterone level but did not impact the level of DHEAS, suggesting that the increase in the pregnancy rate and the reduction in the miscarriage rate may also be associated with lower testosterone levels. However, the DHEAS data were derived only from three small RCTs, so the effect estimate was quite uncertain and needs further evidence.

While infertility, acne and hirsutism secondary to anovulation and hyperandrogenemia jeopardize only reproductive function and affect only the woman's appearance, the metabolic disorders that occur in 37% of patients with PCOS are more detrimental to health [63]. We selected two indicators directly related to insulin resistance and obesity, namely, the fasting insulin level and BMI, to evaluate the efficacy of acupuncture and moxibustion for treating metabolic disorders, and both methods showed a positive result. Previous meta-analyses have also supported the benefits of acupuncture and related techniques for treating metabolic disorders such as type 2 diabetes mellitus and obesity [12, 13]. This consistency increases the credibility of our findings.

In subgroup analyses, we found multiple significant subgroup differences in the ovulation rate, LH level, FSH level, LH/FSH ratio, and total testosterone level. Based on the guidance proposed by Sun et al. for assessing the credibility of subgroup effects, some of the subgroup differences are unreliable because they were in the opposite direction to that anticipated or the residual heterogeneity remained high. We are confident that the following subgroup effect claims can be made: warm-needle moxibustion and ≥3 months of treatment are better for reducing the LH/FSH ratio, and ≥3 months of treatment is better for reducing the total testosterone level. Of course, the other significant findings in the subgroup analysis could serve as clues for future studies designed to explore subgroup effects.

A small number of mild adverse events, including subcutaneous bleeding, pain at the acupuncture site, needle sickness, and pelvic pain, were reported in the included studies. Of these, pelvic pain was likely due to the administration of clomiphene, and the other adverse events were likely related to acupuncture. No moxibustion-related adverse events were reported. Based on these safety profiles, acupuncture and moxibustion therapy seems to be safe in patients with PCOS. However, in our clinical experience, the administration of acupuncture and moxibustion for PCOS requires considering contraindications in terms of timing. Specifically, the treatment should be avoided during the first five days of the menstrual period and discontinued during pregnancy to avoid excessive menstrual flow and increasing the risk of miscarriage and preterm delivery, as the effect of acupoint stimulation on these events cannot be completely ruled out [64, 65]; this was also common practice in the studies included in this systematic review (see Table S2 for details).

To our knowledge, this is the first systematic review to assess the effects of acupuncture combined with moxibustion in patients with PCOS, filling an evidence gap for complementary and alternative treatments for PCOS. The methodological strengths of our study included the systematic literature search, the reasonable statistical analysis plan, and the standardized appraisal of quality of evidence, which ensured the comprehensiveness of the body of evidence and the objectivity of the conclusions.

The study has some limitations. The first and main limitation is that most of the included studies had a moderate-to-high risk of bias, which became a reason to reduce the level of evidence for all outcomes. Second, there was substantial interstudy heterogeneity for some outcomes (e.g., FSH, oestradiol) that could not be explained by the prespecified subgroup analyses, and the prediction intervals suggested that a similar new study would yield estimates that significantly differ from the current CIs, making the direction and precision of the effect estimates for these outcomes unstable. Nevertheless, the results estimated by the random-effects model represent the average effect of acupuncture combined with moxibustion on these outcomes and may preliminarily inform an overall effective or ineffective conclusion for clinical practice. Third, tests of publication bias were not performed for some outcomes due to insufficient samples, but we did not downgrade the level of evidence for these outcomes because there was also no evidence to confirm publication bias.

## 5. Conclusion

Among patients with PCOS, the combined use of acupuncture and moxibustion as a complementary therapy to basic treatments has additional efficacy regarding increased pregnancy and ovulation rates and reduced miscarriage rate and can also help improve the LH level, LH/FSH ratio, fasting insulin level, and BMI. No significant effects were found for the levels of FSH, oestradiol, or DHEAS. All adverse events were mild. Based on these findings, we believe that using acupuncture combined with moxibustion rather than acupuncture alone for treating PCOS is an effective and safe approach that is also more in line with real practice in Chinese hospitals.

## Figures and Tables

**Table 1:** Characteristics of the included studies.

Author	Sample size (E/C)	Mean age (E/C, year)	Course of PCOS (E/C, year)	Type and dose of acupuncture and moxibustion	Cointerventions	Course of treatment	Baseline LH (E/C, mIU/mL)	Baseline FSH (E/C, mIU/mL)	Baseline TT (E/C, ng/dL)
Chen 2020	30/30	29.55/29.47	3.10/3.06	MA + WNM; 30 min, q.o.d.	CHM	3 months	NR	NR	NR
Cui 2015	33/33	33.45/31.38	1.43/1.54*∗*	MA + suspended moxibustion; 25 min/session, q.w.	CHM	6 cycles	9.44/9.42	5.42/5.37	51.3/57.6
Gao 2019a	30/30	28.1/27.6	3.9/3.6	MA + WNM; 30 min/session, q.o.d.	EEC + HCG	3 cycles	15.97/16.24	4.83/4.79	49.6/48.7
Gao 2019b	30/30	28.3/27.2	4.2/3.5	MA + WNM; 30 min/session, q.o.d.	EEC + HCG + CHM	3 cycles	16.09/15.45	4.97/4.94	50.1/47.6
He 2020	35/35	31.97/32.05	3.48/3.52	EA 20 min/session + WNM 30 min/session	Clomiphene	NR	37.53/37.13	9.98/9.83	100.0/101.4
Jiang 2020	44/44	31.22/30.12	NR	MA + suspended moxibustion; 20 min/session, q.o.d.	EEC + letrozole + HCG	NR	14.11/13.45	5.99/6.15	35.2/34.9
Li 2019	50/50	27.78/28.03	2.97/2.56	MA + WNM; 20 min/session, q.o.d.	Clomiphene	3 cycles	16.12/16.08	6.82/6.87	NR
Li 2018	30/30	25.70/26.00	3.91/4.30	MA 30 min/session + moxibustion box 15–30 min/session; t.i.w.	CHM	6 cycles	14.15/14.25	3.86/3.45	79/79.1
Lin 2021	35/35	27.74/26.71	3.13/3.18	EA + ginger-separated moxibustion; 30 min/session, q.o.d.	Letrozole	3 cycles	NR	NR	NR
Lv 2016	100/100	32.00/33.00	NR	EA + WNM; dose was not reported	Clomiphene + HCG	NR	14.19/13.96	5.85/6.13	33.1/35.2
Peng 2020	34/34	28.42/28.31	NR	MA + WNM; 30 min/session	Clomiphene + HCG	NR	NR	NR	NR
Qiao 2012	30/30	20–25 y: 7/8	2–3 y: 17/19*∗*	MA + suspended moxibustion; 30 min/session, q.d.	CHM	6 cycles	14.55/14.46	7.09/7.04	81.1/80.9
		26–30 y: 16/16	3–4 y: 8/9						
		31–35 y: 7/6	>4 y: 4/3						
Qiu 2019	38/37	29.07/28.84	4.61/4.42^∗^	MA + WNM; 30 min/session, q.o.d.	Letrozole + HMG	3 cycles	8.61/8.32	6.35/6.29	38.3/36.8
Shangguan 2017	35/35	NR	NR	EA, 15–25 min/session + WNM, 2 cones/session; q.o.d.	Clomiphene	9 months	37.35/35.31	8.89/9.92	107.8/111.0
Wang 2021	31/31	28.40/28.52	3.43/3.37	MA, 30 min/session + thunder-fire moxibustion, 1 cone/session; q.o.d.	Clomiphene + progesterone for amenorrhea	3 months	11.74/11.71	5.69/5.65	62.8/62.2
Wang 2020	30/30	30.36/29.81	NR	MA + WNM; 20 min/session, 10 sessions per month	CHM	3 cycles	31.91/34.85	8.70/9.01	102.9/106.9
Xie 2019	50/50	25.34/25.32	NR	MA 30 min/session + WNM 2 cone/session	Clomiphene + progesterone^#^	3 cycles	14.07/13.98	5.89/6.14	35.7/35.4
Xing 2020	36/36	34.85/34.24	4.64/4.94*∗*	EA + WNM; 30 min/session, t.i.w.	Triptorelin + rhFSH + HCG + IVF	3 cycles	14.97/14.90	5.61/5.44	40.1/40.6
Xu C 2019	30/30	28.87/29.571	2.53/2.83	MA + WNM; 40 min/session, q.o.d.	Clomiphene + progesterone^#^	3 cycles	15.6/14.73	5.83/5.77	91.6/91.9
Xu 2020	41/41	27.73/28.16	4.01/3.71*∗*	MA + WNM; 30 min/session, q.o.d.	Clomiphene + progesterone^#^	9 months	25.6/25.7	9.4/9.1	62.8/63.2
Xu *J* 2019	30/30	27.83/26.17	1.94/1.97	MA + WNM; 30 min/session, q.o.d.	Lifestyle intervention	3 cycles	13.52/13.53	7.95/7.53	23.9/25.4
Yue 2019	30/30	25.17/26.23	2.97/3.20	MA + moxibustion box; 30 min/session, q.o.d.	Letrozole + CHM	3 cycles	14.76/14.54	5.52/5.76	66.6/66.0
Zheng 2019	30/30	28.50/28.63	NR	MA + WNM, 3 cones/session, q.d.	CHM	3 cycles	NR	NR	NR
Zhong H 2019	30/30	26.77/26.57	3.64/3.21	MA + moxibustion box; 30 min/session, b.i.w.	EEC	3 months	14.48/14.60	8.28/8.11	73.9/72.6
Zhong Q 2019	63/63	33 ± 5/33 ± 5	1.50/1.53*∗*	MA 25 min/session, q.w. + suspended moxibustion 15 min/session, q.d.	CHM	6 cycles	9.43/9.46	5.40/5.41	55.3/55.9
Zhu 2015	42/40	28.86/29.02	4.55/4.33*∗*	MA + suspended moxibustion, 20 min/session, q.o.d.	EEC + letrozole + HMG	3 cycles	8.50/8.21	6.24/6.31	39.5/37.9

C/*E* = experimental group/control group; CHM = Chinese herbal medicine; DEE = desogestrel—ethinyl oestradiol; EA = electroacupuncture; EEC = ethinyl oestradiol—cyproterone; FSH = follicle-stimulating hormone; HCG = human chorionic gonadotropin; HMG = human menopausal gonadotropin; IVF = in vitro fertilization; LH = luteinizing hormone; MA = manual acupuncture; NR = not reported; PCOS = polycystic ovary syndrome; rhFSH = recombinant human follicle-stimulating hormone; TT = total testosterone; WNM = needle warming moxibustion; q.d. = once a day; q.o.d. = once every other day; t.i.w. = three times a week; b.i.w. = twice a week; q.w. = once a week. *∗*Data are the course of infertility; ^#^progesterone was only for patients with amenorrhea.

## Data Availability

The data used to support the findings of this study are available from the corresponding author upon request.

## Abbreviations

ASRM: American society for reproductive medicine
BMI: Body mass index
CI: Confidence interval
CINeMA: Confidence in network meta-analysis
DHEAS: Dehydroepiandrosterone sulfate
ESHRE: European society of human reproduction and embryology
FSH: Follicle-stimulating hormone
GRADE: The grading of recommendations, assessment, development, and evaluations
LH: Luteinizing hormone
MD: Mean difference
PCOS: Polycystic ovary syndrome ()
PCOSAct: The PCOS acupuncture and clomiphene trial
RCT: Randomized controlled trial
RR: Risk ratio.

## Supplementary Materials

Supplementary Materials Table S1. Search strategy. Table S2. Details of acupuncture and moxibustion treatment. Table S3. Formula of Chinese herbal medicine. Table S4. Results of subgroup analyses. Table S5. Results of sensitivity analyses. Table S6. GRADE evidence profile.
