# Supplementary material for: Effects of Acupuncture Combined with Moxibustion on Reproductive and Metabolic Outcomes in Patients with Polycystic Ovary Syndrome: A Systematic Review and Meta-Analysis
Source: Evid Based Complement Alternat Med. 2022 Mar 31;2022:3616036. doi: 10.1155/2022/3616036 (PMC8991411; doi:10.1155/2022/3616036)
Supplement: Supplementary Materials — Table S1. Search strategy. Table S2. Details of acupuncture and moxibustion treatment. Table S3. Formula of Chinese herbal medicine. Table S4. Results of subgroup analyses. Table S5. Results of sensitivity analyses. Table S6. GRADE evidence profile. [file 3616036.f1.docx]

**Supplementary files**

Contents

Table S1. Search strategy.

Table S2. Details of acupuncture and moxibustion treatment.

Table S3. Formula of Chinese herbal medicine.

Table S4. Results of subgroup analyses.

Table S5. Results of sensitivity analyses.

Table S6. GRADE evidence profile.

Table S1. Search strategy.

| PubMed | (acupuncture[mh] OR acupuncture therapy[mh] OR auriculotherapy[mh] OR acupuncture[tw] OR auriculotherapy[tw] OR electro-acupuncture[tw] OR electroacupuncture[tw] OR skin needle*[tw] OR filiform needle*[tw] OR fire needle*[tw] OR silver needle*[tw] OR edged needle*[tw] OR intradermal needle*[tw] OR plum-blossom needle*[tw] OR microwave needle*[tw] OR superficial needle*[tw] OR needling[tw] OR acupoint* [tw] OR meridian*[tw] OR auricular point*[tw]) AND (moxibustion[mh] OR moxibustion[tw] OR moxa[tw] OR thunder-fire[tw] OR taiyi[tw] OR tai yi[tw]) AND (polycystic ovary syndrome[mh] OR polycystic ovar*[tw] OR stein-leventhal[tw] OR PCOS[tw]) NOT (animals[mh] NOT humans[mh]) |
| --- | --- |
| EMBASE | (acupuncture/exp OR 'acupuncture therapy'/exp OR auriculotherapy/exp OR acupuncture:ab,ti OR auriculotherapy:ab,ti OR electro-acupuncture:ab,ti OR electroacupuncture:ab,ti OR skin needle*:ab,ti OR 'filiform needle*':ab,ti OR 'fire needle*':ab,ti OR 'silver needle*':ab,ti OR 'edged needle*':ab,ti OR 'intradermal needle*':ab,ti OR 'plum-blossom needle*':ab,ti OR 'microwave needle*':ab,ti OR 'superficial needle*':ab,ti OR needling:ab,ti OR acupoint*:ab,ti OR meridian*:ab,ti OR 'auricular point*':ab,ti) AND (moxibustion/exp OR moxibustion:ab,ti OR moxa:ab,ti OR thunder-fire:ab,ti OR taiyi:ab,ti OR 'tai yi':ab,ti) AND ('polycystic ovary syndrome'/exp OR 'polycystic ovar*':ab,ti OR stein-leventhal:ab,ti OR PCOS:ab,ti) |
| Cochrane Controlled Register of Trials | #1 MeSH descriptor: [acupuncture] explode all trees  #2 MeSH descriptor: [acupuncture therapy] explode all trees  #3 MeSH descriptor: [auriculotherapy] explode all trees  #4 (acupuncture OR acupuncture therapy OR auriculotherapy OR electro-acupuncture OR electroacupuncture OR skin needle* OR filiform needle* OR fire needle* OR silver needle* OR edged needle* OR intradermal needle* OR plum-blossom needle* OR microwave needle* OR superficial needle* OR needling OR acupoint* OR meridian* OR auricular point*):ti,ab,kw  #5 MeSH descriptor: [moxibustion] explode all trees  #6 (moxibustion OR moxa OR thunder-fire OR taiyi OR tai yi):ti,ab,kw  #7 MeSH descriptor: [polycystic ovary syndrome] explode all trees  #8 (polycystic ovar* OR stein-leventhal OR PCOS):ti,ab,kw  #9 (#1 OR #2 OR #3 OR #4) AND (#5 OR #6) AND (#7 OR #8) |
| CNKI | TKA=(针+针刺+电针)*(灸+艾灸+灸法+艾条灸+热敏灸+神针+长蛇灸+隔药灸+隔附子饼灸+隔姜灸+隔盐灸+麦粒灸)*(多囊卵巢综合征+多囊卵巢+stein-leventhal综合征+S-L综合征+PCOS) OR TKA=针灸*(多囊卵巢综合征+多囊卵巢+stein-leventhal综合征+S-L综合征+PCOS)  ***English translation:***  TKA=(needling + acupuncture + electroacupuncture)*(moxibustion + moxibustion therapy +moxa stick moxibustion + heat-sensitive moxibustion + moxa roll + hydra moxibustion + herb-separated moxibustion + aconite cake-separated moxibustion + ginger-separated moxibustion + salt-separated moxibustion + seed-sized moxibustion)*(polycystic ovarian syndrome + polycystic ovary + stein-leventhal syndrome + S-L syndrome + PCOS) OR TKA=acupuncture and moxibustion*(polycystic ovarian syndrome + polycystic ovary + stein-leventhal syndrome + S-L syndrome + PCOS) |
| Wanfang | 主题:(针 or 针刺 or 电针) and (灸 or "艾灸" or "灸法" or "艾条灸" or "热敏灸" or "神针" or "长蛇灸" or "隔药灸" or "隔附子饼灸" or "隔姜灸" or "隔盐灸" or "麦粒灸") and ("多囊卵巢综合征" or "多囊卵巢" or "stein-leventhal综合征" or "S-L综合征" or "PCOS")  ***English translation:***  Subject:(needling or acupuncture or electroacupuncture) and ("moxibustion" or "moxibustion therapy" or "moxa stick moxibustion" or "heat-sensitive moxibustion" or "moxa roll" or "hydra moxibustion" or "herb-separated moxibustion" or "aconite cake-separated moxibustion" or "ginger-separated moxibustion" or "salt-separated moxibustion" or "seed-sized moxibustion") and ("polycystic ovarian syndrome" or "polycystic ovary" or "stein-leventhal syndrome" or "S-L syndrome" or "PCOS") |
| VIP | (U=(针 OR 针刺 OR 电针) AND U=(灸 OR 艾灸 OR 灸法 OR 艾条灸 OR 热敏灸 OR 神针 OR 长蛇灸 OR 隔药灸 OR 隔附子饼灸 OR 隔姜灸 OR 隔盐灸 OR 麦粒灸) AND U=(多囊卵巢综合征 OR 多囊卵巢 OR stein-leventhal综合征 OR S-L综合征 OR PCOS)) OR (U=针灸 AND U=(多囊卵巢综合征 OR 多囊卵巢 OR stein-leventhal综合征 OR S-L综合征 OR PCOS))  ***English translation:***  (U=(needling OR acupuncture OR electroacupuncture) AND U=(moxibustion OR moxibustion therapy OR moxa stick moxibustion OR heat-sensitive moxibustion OR moxa roll OR hydra moxibustion OR herb-separated moxibustion OR aconite cake-separated moxibustion OR ginger-separated moxibustion OR salt-separated moxibustion OR seed-sized moxibustion) AND U=(polycystic ovarian syndrome OR polycystic ovary OR stein-leventhal syndrome OR S-L syndrome OR PCOS)) OR (U=acupuncture and moxibustion AND U=(polycystic ovarian syndrome OR polycystic ovary OR stein-leventhal syndrome OR S-L syndrome OR PCOS)) |
| Chinese Biomedical Literature database | (针刺[主题词] OR 针[全部字段] OR 针刺[全部字段] OR 电针[全部字段]) AND (灸法[主题词] OR 灸[全部字段] OR 艾灸[全部字段] OR 灸法[全部字段] OR 艾条灸[全部字段] OR 热敏灸[全部字段] OR 神针[全部字段] OR 长蛇灸[全部字段] OR 隔药灸[全部字段] OR 隔附子饼灸[全部字段] OR 隔姜灸[全部字段] OR 隔盐灸[全部字段] OR 麦粒灸[全部字段]) AND (多囊卵巢综合征[主题词] OR 多囊卵巢综合征[全部字段] OR 多囊卵巢[全部字段] OR stein-leventhal综合征[全部字段] OR S-L综合征[全部字段] OR PCOS[全部字段])  ***English translation:***  (acupuncture [MeSH Term] OR needling [All Fields] OR acupuncture [All Fields] OR electroacupuncture [All Fields]) AND (moxibustion therapy [MeSH Term] OR moxibustion [All Fields] OR moxibustion [All Fields] OR moxibustion therapy [All Fields] OR moxa stick moxibustion [All Fields] OR heat-sensitive moxibustion [All Fields] OR moxa roll [All Fields] OR hydra moxibustion [All Fields] OR herb-separated moxibustion [All Fields] OR aconite cake-separated moxibustion [All Fields] OR ginger-separated moxibustion [All Fields] OR salt-separated moxibustion [All Fields] OR seed-sized moxibustion [All Fields]) AND (polycystic ovarian syndrome [MeSH Term] OR polycystic ovarian syndrome [All Fields] OR polycystic ovary [All Fields] OR stein-leventhal syndrome [All Fields] OR S-L syndrome [All Fields] OR PCOS [All Fields]) |
| clinicaltrials.gov | Condition or disease: polycystic ovar* OR stein-leventhal OR PCOS  Other terms: (acupuncture OR auriculotherapy OR electro-acupuncture OR electroacupuncture OR needle OR needling OR acupoint* OR meridian* OR auricular point*) AND (moxibustion OR moxa OR thunder-fire OR taiyi OR tai yi)  Study type: interventional studies |
| Chinese Clinical Trial Registry | 疾病名称: 多囊卵巢  干预措施: 针 OR 灸  ***English translation:***  Disease name: polycystic ovarian  Intervention: polycystic ovarian OR moxibustion |
| MedRxiv | acupuncture AND moxibustion AND (polycystic ovar* OR stein-leventhal OR PCOS) |
| bioRxiv | acupuncture AND moxibustion AND (polycystic ovar* OR stein-leventhal OR PCOS) |

Table S2. Details of acupuncture and moxibustion treatment.

| **Author** | **Acupoints for Acupuncture** | **Acupoints for moxibustion** | **Treatment timing**^*^ |
| --- | --- | --- | --- |
| Chen 2020 | CV6, CV12, KI1, CV4, ST36, SP9, KI3, LR3, LI11, LI4, DU24, DU20 | CV4 | Stop on the menstrual cycle day 1-3 |
| Cui 2015 | Before ovulation: CV4, CV3, ST29, ST40, SP6  After ovulation: EX-HN3, KI3, LR3 | ST36 | Start from the menstrual cycle day 5; stop moxibustion during ovulation |
| Gao 2019 | DU20, DU12, GV9, GV4, GV3, EX-B8, BL23 | DU20, DU12, GV9, GV4, GV3, EX-B8, BL23 | Stop during the menstrual period |
| He 2020 | CV4, CV3, CV6, BL32, SP6, EX-CA1 (EA: attached to a group of ipsilateral limb acupoints) | All acupoints expect those for EA | Stop during the menstrual period |
| Jiang 2020 | EX-CA1, CV3, SP6; adding CV12, ST40, and ST29 for intermingled phlegm and blood stasis syndrome; SP8 and KI3 for yin asthenia generating intrinsic heat syndrome; ST36, KI3, and CV4 for deficiency of qi and blood syndrome; LR3 and PC6 for liver depression and blood deficiency | ST29 | During follicular phase and ovulation period |
| Li 2019 | CV6, CV4, EX-CA1, BL23, BL20 | CV6, CV4, EX-CA1, BL23, BL20 | Not reported |
| Li 2018 | Supine position: DU20, CV4, CV3, GV4, SP4, KI6  Prone position: GV4 | Follicullar phase: CV4  Luteal phase: GV4 | Stop during menstrual period  Discontinue when pregnancy |
| Lin 2021 | Menstrual period: EX-CA, ST29, SP6, LR3, LI4 (EA: attached to EX-CA1 and ST29)  Follicullar phase: CV6, KI12 (EA), Extra ovary (EA), SP6 (EA), ST36 (EA), KI3  Ovulatory period: Extra ovary (EA), GB27 (EA), CV3 (EA), SP10 (EA), SP6 (EA), LI4, LR3  Luteal phase: EX-CA1 (EA), SP10 (EA), ST36 (EA), SP6, KI3 | Follicullar phase: CV4 CV4, CV8  Luteal phase: CV4 | Discontinue when pregnancy |
| Lv 2016 | Menstrual period: EX-CA1, CV4, ST29, CV3  Follicullar phase: EX-CA1, CV3  Ovulatory period: EX-CA1, ST28 (EA)  Luteal phase: EX-CA1 | Follicullar phase: EX-CA1  Luteal phase: EX-CA1 | Stop acupuncture for eumenorrhea and use only acupuncture for hypomenorrhea during the menstrual period |
| Peng 2020 | EX-CA1, CV4, CV3, SP10, CV6  Ovulatory period: EX-CA1 (EA), ST28 | Follicullar phase: EX-CA1, CV3 | Not reported |
| Qiao 2012 | CV3, CV4, EX-CA1, BL23, SP6, ST40 | CV4, SP6 | Stop during menstrual period  Discontinue when pregnancy |
| Qiu 2019 | EX-CA1, SP6, CV3; adding SP10 and LR3 for liver depression and blood deficiency syndrome; ST40 and ST29 for intermingled phlegm and blood stasis syndrome; CV4 and ST36 for yin asthenia generating intrinsic heat syndrome; BL23 and HT7 for kidney deficiency and insomnia | Guiyuan, CV6 | Start from the first day of treatment |
| Shangguan 2017 | CV4, CV6, CV3, SP6, BL32, EX-CA1; adding  LR3, LI4, LR14, and SP10 for qi-stagnation and blood stasis syndrome; KI14, SP9, and ST40 for phlegm-dampness block syndrome; BL18, BL23, and GV3 for insufficient liver and kidney syndrome; BL20, CV12, CV10, SP10, ST36 for asthenia of qi and blood syndrome (EA: attached to a group of ipsilateral limb acupoints) | All acupoints expect those for EA | Start from the menstrual cycle day 5 |
| Wang 2021 | ST25, CV4, CV3, EX-CA1, KI13, SP10, ST36, SP6, KI7 | CV4, CV6, ST36, SP10, SP6 | Not reported |
| Wang 2020 | CV12, CV9, CV6, CV4, ST29, EX-CA1, TE6, LI4, ST36, SP6 | CV6, CV4, EX-CA1 | Stop during menstrual period |
| Xie 2019 | CV3, EX-CA1, BL23, SP10; adding ST40 and CV12 for intermingled phlegm and blood stasis syndrome; KI3 and SP8 hyperactivity of fire due to yin deficiency; CV4 and ST36 for asthenia of qi and blood | The same as acupuncture | Start from the menstrual cycle day 5 |
| Xing 2020 | Menstrual period: LI4, BL18, BL23, BL32  Follicullar phase: KI16, EX-CA1, ST36, CV4, KI2, SI3  Ovulatory period: ST28 (EA), ST29 (EA), CV3, LI4, ST36, CV4, LR3, scalp acupuncture at MS4  Luteal phase: GV9, BL17, BL23, LU7, KI6  Adding CV4 and KI3 for kidney deficiency syndrome; SP10 and BL17 for blood stasis syndrome; ST40 and CV12 for phlegm-dampness syndrome; BL20 and SP9 for spleen deficiency syndrome; LR3 and BL18 for liver depression syndrome | Ovulatory period: acupoints were not mentioned  Luteal phase: BL23, BL31, BL32, BL33, BL34 | From the second menstrual period after enrollment  Menstrual period: adding moxibustion for hypomenorrhea (acupoints were not mentioned) |
| Xu CX 2019 | CV6, CV4, EX-CA1, SP10, SP6, SP9, SP8, ST40, KI3, LR3 | CV4, CV6, EX-CA1 | From the day after the end of the cycle to the next cycle comes |
| Xu 2020 | CV12, CV4, CV6, CV3, GV4, GV3, GV2 | CV4, CV6, GV4, GV3, GV2 | Start from menstrual period day 5 |
| Xu JM 2019 | Main: SP6, CV4, CV3, EX-CA1, KI2; adding SP10 for blood stasis syndrome; ST40 for phlegm-dampness syndrome | At the main acupoints | Treatment was carried out two months/cycles before menstruation |
| Yue 2019 | Group 1: CV4, CV6, EX-CA1, KI3, ST40, ST36, SP6, ST29, SP9  Group 2: CV4, CV3, EX-CA1, LI4, LR3 | CV6, CV4, EX-CA1, ST29 | Start from the menstrual cycle day 5 and stop when ovulation or after last ultrasound monitoring for patients without ovulation |
| Zheng 2019 | CV4, SP6, EX-CA1, ST40 | CV4, SP6, EX-CA1, ST40 | Start from the menstrual cycle day 5 to day 22 |
| Zhong H 2019 | BL31, BL32, BL33, BL34 | BL31, BL32, BL33, BL34 | Stop from the menstrual cycle starts to three days after the end of menstruation |
| Zhong QZ 2019 | EX-HN3, CV4, LR3, CV3, KI3, ST29, SP6, ST40 | ST36 | Start from the menstrual cycle day 5 |
| Zhu 2015 | EX-CA1, SP6, CV3; adding ST29, ST40 and CV12 for intermingled phlegm and blood stasis syndrome; KI3 and SP8 hyperactivity of fire due to yin deficiency; CV4 and ST36 for asthenia of qi and blood; PC6 and LR3 for blood stasis and liver depression syndrome | ST29 | Start from the first menstrual cycle |

^*^ The withdrawal bleeding caused by progesterone was treated as the start the menstrual cycle for patients without menstruation. EA: electroacupuncture

Table S3. Formula of Chinese herbal medicine.

| **Study** | **Name** | Component and dose |
| --- | --- | --- |
| Chen 2020 | Bu Shen Jian Pi Qu Tan decoction (*补肾健脾祛痰汤*) | Lycii Fructus, Ligustri Lucidi Fructus, Rehmanniae Radix Praeparata, Poria, Salviae Miltiorrhizae Radix et Rhizoma (each 15 g), Cuscutae Semen, Astragali Radix (each 20 g), Atractylodis Rhizoma, Atractylodis Macrocephalae Rhizoma, Arisaema Cum Bile, Codonopsisradix, Nelumbinis Folium, Pinelliae Rhizoma Praeparatum, Alismatis Rhizoma (each 10 g), Glycyrrhizae Radix et Rhizoma Praeparata Cum Melle (each 6 g). Adding Citri Sarcodactylis Fructus for nausea, saposhnikoviae Radix for diarrhea, and Bupleuri Radix for depressure. |
| Cui 2015 | Bu Shen Hua Tan Tiao Zhou granules (*补肾化痰调周颗粒*) | 1) Menstrual period: Linderae Radix, Corydalis yanhusuo (each 15g), Prepared Cyperi Rhizoma, Moutan Cortex, Crataegi Fructus, Salviae Miltiorrhizae Radix et Rhizoma, Paeoniae Radix Rubra, Faeces Trogopterori, Leonur Iherba, Poria10g (each 10g)  2) Follicullar phase: Stir-fried Angelicae Sinensis Radix, Moutan Cortex, Paeoniae Radix Alba, Huai Dioscoreae Rhizoma, Corni Fructus, Poria, Alismatis Rhizoma, Himalayan Teasel Root, Cuscutae Semen, Citri Reticulatae Pericarpium, Prepared Pinelliae Rhizoma, Prepared Cyperi Rhizoma (each 10g), Codonopsisradix, Atractylodis Rhizoma (each 15g),  3) Ovulatory period: Bupleuri Radix 6g, Cinnamomi Ramulus, Vinegar-baked Angelicae Sinensis Radix, Salviae Miltiorrhizae Radix et Rhizoma, Paeoniae Radix Rubra, Moutan Cortex, Poria, Himalayan Teasel Root, Stir-fried Coicis Semen, Carthami Flos, Chuanxiong Rhizoma (each 10g), Dioscoreae Rhizoma, Liquidambaris Fructus (each 15g), Spatholobi Caulis 20g  4) Luteal phase: Codonopsisradix, Paeoniae Radix Alba, Epimedii Folium, Poria, Cervi Cornu, Citri Reticulatae Pericarpium, Prepared Pinelliae Rhizoma, Acori Tatarinowii Rhizoma, Stewed Aucklandiae Radix (each 10g), Atractylodis Rhizoma, Dioscoreae Rhizoma, Himalayan Teasel Root (each 15g), Fluoritum 20g |
| Gao 2019b | Bu Shen Tiao Jing decoction (*补肾调经汤*) | Fluoritum, Dioscoreae Rhizoma (each 30g), Epimedii Folium, Moutan Cortex (each 15g), Cuscutae Semen, Lycii Fructus, Astragali Radix (each 20g), Dipsaci Radix, Angelicae Sinensis Radix, Curcumae Rhizoma, Cyathulae Radix, Cyperi Rhizoma (each 10g), Zanthoxyli Pericarpium 6g |
| Li 2018 | You Gui pills (*右归丸*) | Rehmanniae Radix Praeparata, Dioscoreae Rhizoma, Corni Fructus, Lycii Fructus, Cuscutae Semen, Cervi Cornus Colla, Eucommiae Cortex, Cinnamomi Cortex, Angelicae Sinensis Radix, Aconiti Lateralis Radix Praeparata; the dose was not reported |
| Qiao 2012 | Chinese Medicine Sequential Therapy | 1) Follicullar phase: Gui Shao Di Huang decoction (*归芍地黄汤*): Salviae Miltiorrhizae Radix et Rhizoma, Paeoniae Radix Alba, Rehmanniae Radix, Rehmanniae Radix Praeparata, Huai Dioscoreae Rhizoma, Corni Fructuslog, Poria, Moutan Cortex, Stir-baked Trionycis Carapax, Ligustri Lucidi Fructus, Himalayan Teasel Root, Visciherba, Prunellae Spica (each 10g), Puerariae Lobatae Radix, Coicis Semen (each 20g), Guangdong Citri Reticulatae Pericarpium 9g, Coptidis Rhizoma 3g  2) Ovulatory period: Bu Shen Cu Pai Luan decoction (*补肾促排卵汤*): Salviae Miltiorrhizae Radix et Rhizoma, Moutan Cortex, Paeoniae Radix Rubra, Paeoniae Radix Alba, Rehmanniae Radix Praeparata, Corni Fructus, Huai dioscoreae Rhizoma, Poria, Himalayan Teasel Root, Visciherba, Cuscutae Semen, Angelicae Sinensis Radix, Stir-fried Faeces Trogopterori, Atractylodis Rhizoma (each 10g), Coicis Semen, Puerariae Lobatae Radix (each 20g) Carthami Flos 6g, Guangdong Citri Reticulatae Pericarpium 9g  3) Luteal phase: Yu Lin Zhu (*毓麟珠*): Salviae Miltiorrhizae Radix et Rhizoma, Moutan Cortex, Paeoniae Radix Rubra, Paeoniae Radix Alba, Huai dioscoreae Rhizoma, Himalayan Teasel Root, Visciherba, Fluoritum, Leonuri Fructus, Atractylodis Rhizoma, Prunellae Spica, Vaccariae Semen, Cyperi Rhizoma (each 10g), Coicis Semen, Puerariae Lobatae Radix (each 20g), Carthami Flos 6g, Guangdong Citri Reticulatae Pericarpium 9g, Coptidis Rhizoma 3g  4) Menstrual period: Wu Wei Tiao Jing decoction (*五味调经汤*): Salviae Miltiorrhizae Radix et Rhizoma, Paeoniae Radix Rubra, Stir-fried Angelicae Sinensis Radix, Lycopi Herba, Stir-fried Faeces Trogopterori, Prepared pollen of Longbract Cattail, Vaccariae Semen, Platycladi Semen, Himalayan Teasel Root, Prepared Cyperi Rhizoma, Prepared Atractylodis Rhizoma (each 10g), Leonur Iherba, Cyathulae Radix (each 15g), Puerariae Lobatae Radix, Coicis Semen, Prunellae Spicalog, Dianthi Herba (each 20g), Guangdong Citri Reticulatae Pericarpium 9g |
| Wang 2020 | Cang Fu Dao Tan decoction (*苍附导痰汤*) | Atractylodis Rhizoma, Polygalae Radix, Acori Tatarinowii Rhizoma, Chuanxiong Rhizoma, Glycyrrhizae Radix et Rhizoma, Aurantii Fructus (each 10g), Morindae Officinalis Radix, Cistanches Herba, Cyperi Rhizoma, Citri Reticulatae Pericarpium, Achyranthis Bidentatae Radix, Plantaginis Semen, Angelicae Sinensis Radix, Gleditsiae Spina (each 15g), Poria, Epimedii Folium, Spatholobi Caulis, Leonur Iherba (each 25g), Pinelliae Rhizoma 9g |
| Yue 2019 | Yang Jing Dao Tan decoction (*养精导痰汤*) | Lycii Fructus, Dioscoreae Rhizoma, Corni Fructus, Angelicae Sinensis Radix Rehmanniae Radix Praeparata, Stir-fried Atractylodis Macrocephalae Rhizoma, Lycopi Herba, Pinelliae Rhizoma Praeparatum, Citri Reticulatae Pericarpium, Cyperi Rhizoma (each 10g), Semen Sinapis, Glycyrrhizae Radix et Rhizoma (each 6g), Coicis Semen 20g, Cuscutae Semen 15g |
| Zheng 2019 | Cang Fu Dao Tan decoction (*苍附导痰汤*) | Atractylodis Rhizoma, Cyperi Rhizoma, Citri Reticulatae Pericarpium, Arisaema Cum Bile, Aurantii Fructus, Pinelliae Rhizoma Praeparatum, Poria, Massa Medicata Fermentata, Angelicae Sinensis Radix, Arecae Pericarpium, Cyathulae Radix, Glycyrrhizae Radix et Rhizoma Praeparata Cum Melle; the dose was not reported |
| Zhong QZ 2019 | Tiao Zhou method (*调周法*) | 1) Menstrual period: Corydalis yanhusuo, Linderae Radix (each 15g), Poria, Prepared Cyperi Rhizoma, Leonur Iherba, Moutan Cortex, Faeces Trogopterori, Crataegi Fructus, Paeoniae Radix Rubra, Salviae Miltiorrhizae Radix et Rhizoma (each 10g)  2) Follicullar phase: Atractylodis Rhizoma, Codonopsisradix (each 15g), Stir-fried angelicae Sinensis Radix, Prepared Cyperi Rhizoma, Moutan Cortex, Prepared pinelliae Rhizoma, Paeoniae Radix Alba, Citri Reticulatae Pericarpium, Huai Dioscoreae Rhizoma, Cuscutae Semen, Corni Fructus, Himalayan Teasel Root, Poria, Alismatis Rhizoma (each 15g)  3) Ovulatory period Spatholobi Caulis 20g, Vinegar-baked Bupleuri Radix 6g, Liquidambaris Fructus, Dioscoreae Rhizoma (each 15g), Cinnamomi Ramulus, Chuanxiong Rhizoma, Angelicae Sinensis Radix, Carthami Flos, Salviae Miltiorrhizae Radix et Rhizoma, Stir-fried Coicis Semen, Paeoniae Radix Rubra, Himalayan Teasel Root, Moutan Cortex, Poria (each 10g)  4) Luteal phase: Fluoritum 20g, Atractylodis Rhizoma, Himalayan Teasel Root, Dioscoreae Rhizoma (each 15g), Codonopsisradix, Acori Tatarinowii Rhizoma, Paeoniae Radix Alba, Prepared Pinelliae Rhizoma, Epimedii Folium, Citri Reticulatae Pericarpium, Poria, Cervi Cornu (each 10g) |

Table S4. Results of subgroup analyses.

| **Outcome** | **Type of subgroup** | **No. of studies** | **Effect size (95% CI)** | **I^2^** | **Interaction P value (Residual I^2^) ^*^** |
| --- | --- | --- | --- | --- | --- |
| Pregnancy rate | All studies | 18 | RR 1.81 (1.58, 2.08) | 0% |  |
|  | Basic treatment |  |  |  |  |
|  | WM | 5 | RR 1.51 (1.12, 2.04) | 0% | P=0.07 |
|  | CHM | 11 | RR 2.00 (1.70, 2.36) | 0% |  |
|  | WM+CHM | 2 | RR 1.25 (0.79, 1.98) | 0% |  |
|  | Type of acupuncture |  |  |  |  |
|  | Electronic | 13 | RR 1.71 (1.44, 2.04) | 0% | P=0.31 |
|  | Manual | 5 | RR 1.98 (1.59, 2.47) | 0% |  |
|  | Type of moxibustion |  |  |  |  |
|  | WNM | 8 | RR 1.66 (1.31, 2.11) | 0% | P=0.39 |
|  | Others | 10 | RR 1.89 (1.60, 2.23) | 0% |  |
|  | Course of treatment |  |  |  |  |
|  | ≤ 3 months | 5 | RR 1.50 (1.16, 1.95) | 0% | P=0.21 |
|  | > 3 months | 10 | RR 1.84 (1.53, 2.21) | 0% |  |
| Ovulation rate | All studies | 14 | RR 1.31 (1.22, 1.40) | 18.9% |  |
|  | Basic treatment |  |  |  |  |
|  | WM | 3 | RR 1.61 (1.39, 1.87) | 0.0% | P=0.01 (0%) |
|  | CHM | 9 | RR 1.27 (1.18, 1.37) | 0.0% |  |
|  | WM+CHM | 2 | RR 1.18 (1.03, 1.35) | 0.0% |  |
|  | Type of acupuncture |  |  |  |  |
|  | Electronic | 12 | RR 1.30 (1.20, 1.40) | 13.5% | P=0.46 |
|  | Manual | 2 | RR 1.52 (1.00, 2.30) | 66.5% |  |
|  | Type of moxibustion |  |  |  |  |
|  | WNM | 6 | RR 1.40 (1.24, 1.57) | 18.0% | P=0.11 |
|  | Others | 8 | RR 1.25 (1.15, 1.35) | 0.0% |  |
|  | Course of treatment |  |  |  |  |
|  | ≤ 3 months | 4 | RR 1.45 (1.20, 1.76) | 59.3% | P=0.14 |
|  | > 3 months | 7 | RR 1.24 (1.13, 1.35) | 0.0% |  |
| Miscarriage rate | All studies | 8 | RR 0.45 (0.28, 0.73) | 0% |  |
|  | Basic treatment |  |  |  |  |
|  | WM | 7 | RR 0.47 (0.27, 0.83) | 0.0% | P=0.81 |
|  | CHM | 1 | RR 0.41 (0.17, 0.99) | NA |  |
|  | Type of acupuncture |  |  |  |  |
|  | Electronic | 6 | RR 0.43 (0.24, 0.74) | 0.0% | P=0.86 |
|  | Manual | 2 | RR 0.50 (0.10, 2.44) | 64.3% |  |
|  | Type of moxibustion |  |  |  |  |
|  | WNM | 6 | RR 0.48 (0.29, 0.81) | 0.0% | P=0.57 |
|  | Others | 2 | RR 0.32 (0.09, 1.17) | 6.3% |  |
|  | Course of treatment |  |  |  |  |
|  | ≤ 3 months | 7 | RR 0.48 (0.29, 0.81) | 0.0% | P=0.53 |
|  | > 3 months | 1 | RR 0.31 (0.09, 1.08) | NA |  |
| LH (mIU/mL) | All studies | 22 | MD -2.31 (-2.86, -1.77) | 75.8% |  |
|  | Basic treatment |  |  |  |  |
|  | WM | 5 | MD -1.73 (-3.13, -0.33) | 63.4% | P=0.01 (75.1%) |
|  | CHM | 14 | MD -2.63 (-3.22, -2.05) | 71.4% |  |
|  | WM+CHM | 2 | MD -1.17 (-1.83, -0.52) | 0.0% |  |
|  | Lifestyle intervention | 1 | MD -2.55 (-5.54, 0.44) | NA |  |
|  | Type of acupuncture |  |  |  |  |
|  | Electronic | 18 | MD -2.00 (-2.48, -1.51) | 71.4% | P=0.01 (73.4%) |
|  | Manual | 4 | MD -4.95 (-7.17, -2.74) | 78.2% |  |
|  | Type of moxibustion |  |  |  |  |
|  | WNM | 9 | MD -1.73 (-2.37, -1.08) | 76.7% | P=0.03 (74.6%) |
|  | Others | 13 | MD -2.90 (-3.76, -2.05) | 66.0% |  |
|  | Course of treatment |  |  |  |  |
|  | ≤ 3 months | 5 | MD -1.93 (-3.87, 0.00) | 72.8% | P=0.66 |
|  | > 3 months | 14 | MD -2.39 (-3.06, -1.72) | 75.8% |  |
| FSH (mIU/mL) | All studies | 22 | MD -0.08 (-0.36, 0.21) | 81.0% |  |
|  | Basic treatment |  |  |  |  |
|  | WM | 5 | MD -0.53 (-1.03, -0.03) | 60.8% | P=0.01 (81.5%) |
|  | CHM | 14 | MD 0.03 (-0.31, 0.37) | 83.7% |  |
|  | WM+CHM | 2 | MD 0.50 (0.08, 0.92) | 0.0% |  |
|  | Lifestyle intervention | 1 | MD -0.91 (-2.15, 0.33) | NA |  |
|  | Type of acupuncture |  |  |  |  |
|  | Electronic | 18 | MD -0.16 (-0.43, 0.10) | 74.8% | P=0.30 |
|  | Manual | 4 | MD 0.38 (-0.63, 1.39) | 79.3% |  |
|  | Type of moxibustion |  |  |  |  |
|  | WNM | 9 | MD -0.11 (-0.37, 0.15) | 59.1% | P=0.86 |
|  | Others | 13 | MD -0.06 (-0.54, 0.42) | 86.6% |  |
|  | Course of treatment |  |  |  |  |
|  | ≤ 3 months | 5 | MD -0.36 (-0.60, -0.12) | 16.5% | P=0.14 |
|  | > 3 months | 14 | MD -0.02 (-0.41, 0.36) | 80.8% |  |
| LH/FSH ratio | All studies | 9 | MD -0.47 (-0.64, -0.30) | 67.4% |  |
|  | Basic treatment |  |  |  |  |
|  | WM | 5 | MD -0.63 (-0.75, -0.51) | 26.3% | P<0.01 (0%) |
|  | CHM | 2 | MD -0.35 (-0.56, -0.13) | 0.0% |  |
|  | WM+CHM | 1 | MD -0.25 (-0.39, -0.12) | NA |  |
|  | Lifestyle intervention | 1 | MD -0.26 (-0.66, 0.14) | NA |  |
|  | Type of acupuncture |  |  |  |  |
|  | Electronic | 5 | MD -0.28 (-0.39, -0.17) | 0.0% | P<0.01 (0%) |
|  | Manual | 4 | MD -0.80 (-1.15, -0.46) | 32.2% |  |
|  | Type of moxibustion |  |  |  |  |
|  | WNM | 7 | MD -0.59 (-0.70, -0.48) | 29.9% | P<0.01 (0%) |
|  | Others | 2 | MD -0.26 (-0.38, -0.15) | 0.0% |  |
|  | Course of treatment |  |  |  |  |
|  | ≤ 3 months | 6 | MD -0.30 (-0.40, -0.19) | 28.5% | P=0.04 (0%) |
|  | > 3 months | 1 | MD -1.15 (-1.96, -0.34) | NA |  |
| Total testosterone | All studies | 21 | MD -7.04 (-9.38, -4.70) | 89.2% |  |
| (ng/dl) | Basic treatment |  |  |  |  |
|  | WM | 5 | MD -7.78 (-15.82, 0.26) | 89.7% | P=0.11 |
|  | CHM | 13 | MD -7.07 (-9.50, -4.64) | 91.2% |  |
|  | WM+CHM | 2 | MD -6.04 (-10.96, -1.11) | 0.0% |  |
|  | Lifestyle intervention | 1 | MD -2.59 (-5.42, 0.23) | NA |  |
|  | Type of acupuncture |  |  |  |  |
|  | Electronic | 17 | MD -6.78 (-9.56, -3.99) | 89.6% | P=0.53 |
|  | Manual | 4 | MD -8.36 (-12.44, -4.28) | 74.6% |  |
|  | Type of moxibustion |  |  |  |  |
|  | WNM | 9 | MD -7.04 (-11.51, -2.57) | 84.1% | P=0.95 |
|  | Others | 12 | MD -6.87 (-9.46, -4.29) | 89.6% |  |
|  | Course of treatment |  |  |  |  |
|  | ≤ 3 months | 5 | MD -1.43 (-2.14, -0.73) | 0.0% | P<0.01 (80.4%) |
|  | > 3 months | 13 | MD -8.84 (-12.17, -5.51) | 81.9% |  |
| Oestradiol (pg/mL) | All studies | 15 | MD 2.94 (-1.05, 6.93) | 86.1% |  |
|  | Basic treatment |  |  |  |  |
|  | WM | 4 | MD 2.46 (-0.79, 5.70) | 38.1% | P=0.34 |
|  | CHM | 9 | MD 4.06 (-2.46, 10.58) | 91.5% |  |
|  | WM+CHM | 2 | MD 0.04 (-2.38, 2.47) | 0.0% |  |
|  | Type of acupuncture |  |  |  |  |
|  | Electronic | 13 | MD 4.10 (-0.09, 8.28) | 85.3% | P=0.12 |
|  | Manual | 2 | MD -3.85 (-12.88, 5.17) | 89.9% |  |
|  | Type of moxibustion |  |  |  |  |
|  | WNM | 7 | MD 1.56 (-0.25, 3.37) | 0.0% | P=0.45 |
|  | Others | 8 | MD 4.41 (-2.83, 11.65) | 92.6% |  |
|  | Course of treatment |  |  |  |  |
|  | ≤ 3 months | 4 | MD 6.38 (-1.69, 14.46) | 89.9% | P=0.40 |
|  | > 3 months | 9 | MD 2.14 (-3.62, 7.89) | 88.2% |  |
| Body mass index | All studies | 10 | MD -1.78 (-2.53, -1.03) | 71.1% |  |
| (kg/m^2^) | Basic treatment |  |  |  |  |
|  | WM | 4 | MD -2.03 (-3.92, -0.15) | 80.7% | P=0.81 |
|  | CHM | 4 | MD -1.45 (-2.35, -0.56) | 60.2% |  |
|  | WM+CHM | 2 | MD -1.80 (-2.89, -0.70) | 66.9% |  |
|  | Type of moxibustion |  |  |  |  |
|  | WNM | 4 | MD -1.83 (-2.66, -0.99) | 53.5% | P=0.89 |
|  | Others | 6 | MD -1.72 (-2.96, -0.48) | 79.6% |  |
|  | Course of treatment |  |  |  |  |
|  | ≤ 3 months | 8 | MD -1.51 (-2.05, -0.97) | 39.5% | P=0.45 |
|  | > 3 months | 2 | MD -2.84 (-6.25, 0.57) | 86.5% |  |

CHM = Chinese herbal medicine; FSH = follicle-stimulating hormone; LH = luteinizing hormone; MD = mean difference; NA = not available; RR = risk ratio; WM = western medicine

* Residual I^2^ was only available for subgroups with a significant interaction p value.

Table S5. Results of sensitivity analyses.

| **Outcome** | **Type of subgroup** | **No. of studies** | **No. of patients** | | **Effect size (95% CI)** | **I^2^** |
| --- | --- | --- | --- | --- | --- | --- |
|  |  |  | **Experimental** | **Control** |  |  |
| Pregnancy rate | Main analysis | 18 | 692 | 694 | RR 1.81 (1.58, 2.08) | 0% |
|  | Excluding studies with high risk of bias | 11 | 415 | 412 | RR 1.77 (1.50, 2.10) | 0% |
|  | Fixed effect model | 18 | 692 | 694 | RR 1.85 (1.61, 2.13) | 0% |
| Ovulation rate | Main analysis | 14 | 807 | 801 | RR 1.31 (1.22, 1.40) | 18.9% |
|  | Excluding studies with high risk of bias | 9 | 509 | 500 | RR 1.35 (1.22, 1.51) | 37.6% |
|  | Excluding studies analyzing the number of menstrual cycles | 12 | 532 | 529 | RR 1.27 (1.19, 1.36) | 0% |
|  | Fixed effect model | 14 | 807 | 801 | RR 1.37 (1.28, 1.46) | 18.9% |
| Miscarriage rate | Main analysis | 8 | 191 | 101 | RR 0.45 (0.28, 0.73) | 0% |
|  | Excluding studies with high risk of bias | 7 | 170 | 90 | RR 0.48 (0.29, 0.81) | 0% |
|  | Fixed effect model | 8 | 191 | 101 | RR 0.46 (0.29, 0.74) | 0% |
| Ovarian volume (cm^3^) | Main analysis | 4 | 149 | 149 | MD -0.75 (-1.30, -0.20) | 20.4% |
|  | Excluding studies with high risk of bias | 1 | 50 | 50 | MD -0.70 (-1.59, 0.19) | NA |
| LH (mIU/mL) | Main analysis | 22 | 862 | 858 | MD -2.31 (-2.86, -1.77) | 75.8% |
|  | Excluding studies with high risk of bias | 13 | 489 | 485 | MD -2.37 (-3.19, -1.56) | 75.4% |
| FSH (mIU/mL) | Main analysis | 22 | 862 | 858 | MD -0.08 (-0.36, 0.21) | 81.0% |
|  | Excluding studies with high risk of bias | 13 | 489 | 485 | MD -0.09 (-0.51, 0.33) | 81.4% |
| LH/FSH ratio | Main analysis | 9 | 352 | 353 | MD -0.47 (-0.64, -0.30) | 67.4% |
|  | Excluding studies with high risk of bias | 4 | 124 | 124 | MD -0.46 (-0.73, -0.18) | 42.3% |
| Total testosterone | Main analysis | 21 | 812 | 808 | MD -7.04 (-9.38, -4.70) | 89.2% |
| (ng/dl) | Excluding studies with high risk of bias | 12 | 439 | 435 | MD -7.65 (-10.21, -5.08) | 69.6% |
| Oestradiol (pg/mL) | Main analysis | 15 | 642 | 639 | MD 2.94 (-1.05, 6.93) | 86.1% |
|  | Excluding studies with high risk of bias | 9 | 369 | 365 | MD 2.17 (-3.67, 8.02) | 88.2% |
| Fasting insulin | Main analysis | 4 | 175 | 173 | MD -2.48 (-3.85, -1.12) | 67.8% |
| (mIU/L) | Excluding studies with high risk of bias | 3 | 145 | 144 | MD -3.19 (-4.11, -2.27) | 0% |
| Body mass index | Main analysis | 10 | 373 | 369 | MD -1.78 (-2.53, -1.03) | 71.1% |
| (kg/m^2^) | Excluding studies with high risk of bias | 7 | 285 | 281 | MD -1.92 (-2.96, -0.89) | 79.3% |

CHM = Chinese herbal medicine; DHEAS = dehydroepiandrosterone sulfate; FSH = follicle-stimulating hormone; LH = luteinizing hormone; MD = mean difference; RR = risk ratio; WM = western medicine

* Residual I^2^ was only available for subgroups with a significant interaction p value.

Note: The table does not show unavailable sensitivity analyses.

Table S6. GRADE evidence profile.

| **Quality assessment** | | | | | | | **No of patients** | | **Effect estimate** | | **Quality** | **Importance** |
| --- | --- | --- | --- | --- | --- | --- | --- | --- | --- | --- | --- | --- |
| **No of studies** | **Design** | **Risk of bias** | **Inconsistency** | **Indirectness** | **Imprecision** | **Publication bias** | **Experimental** | **Control** | **Point estimate**  **(95% CI)** | **95% Prediction interval** |  |  |
| **Pregnancy rate (course of treatment: median 3.0 months/cycles)** | | | | | | | | | | | | |
| 18 | RCT | Serious^1^ | No serious inconsistency | No serious indirectness | No serious imprecision | Undetected | 692 | 694 | RR 1.81 (1.58, 2.08) | (1.56, 2.10) | MODERATE | CRITICAL |
| **Ovulation rate (course of treatment: median 3.0 months/cycles)** | | | | | | | | | | | | |
| 14 | RCT | Serious^1^ | No serious inconsistency^3^ | No serious indirectness | No serious imprecision | Undetected | 807 | 801 | RR 1.31 (1.22, 1.40) | (1.14, 1.51) | MODERATE | CRITICAL |
| **Miscarriage rate (course of treatment: median 3.0 months/cycles)** | | | | | | | | | | | | |
| 8 | RCT | Serious^1^ | No serious inconsistency | No serious indirectness | No serious imprecision | Undetected^4^ | 191 | 101 | RR 0.45 (0.28, 0.73) | (0.25, 0.82) | MODERATE | CRITICAL |
| **Ovarian volume (course of treatment: median 4.5 months/cycles; measured with: cm^3^)** | | | | | | | | | | | | |
| 4 | RCT | Very serious^1^ | Serious^2^ | No serious indirectness | No serious imprecision | Undetected^4^ | 149 | 149 | MD -0.75 (-1.30, -0.20) | (-2.49, 0.97) | VERY LOW | IMPORTANT |
| **Luteinizing hormone (course of treatment: median 3.0 months/cycles; measured with: mIU/mL)** | | | | | | | | | | | | |
| 22 | RCT | Serious^1^ | No serious inconsistency | No serious indirectness | No serious imprecision | Undetected | 862 | 858 | MD -2.31 (-2.86, -1.77) | (-4.54, -0.09) | MODERATE | IMPORTANT |
| **Follicle-stimulating hormone (course of treatment: median 3.0 months/cycles; measured with:** **mIU/mL)** | | | | | | | | | | | | |
| 22 | RCT | Serious^1^ | Very serious^2^ | No serious indirectness | No serious imprecision | Undetected | 862 | 858 | MD -0.08 (-0.36, 0.21) | (-1.33, 1.17) | VERY LOW | IMPORTANT |
| **Luteinizing hormone to follicle-stimulating hormone ratio (course of treatment: median 3.0 months/cycles; measured with: ratio)** | | | | | | | | | | | | |
| 9 | RCT | Serious^1^ | No serious inconsistency | No serious indirectness | No serious imprecision | Undetected^4^ | 352 | 353 | MD -0.47 (-0.64, -0.30) | (-0.92, -0.01) | MODERATE | IMPORTANT |
| **Total testosterone (course of treatment: median 3.0 months/cycles; measured with:** **ng/dl) 5** | | | | | | | | | | | | |
| 21 | RCT | Serious^1^ | Serious^2^ | No serious indirectness | No serious imprecision | Suspected^5^ | 812 | 808 | MD -7.04 (-9.38, -4.70) | (-17.12, 3.04) | VERY LOW | IMPORTANT |
| **Dehydroepiandrosterone sulfate (course of treatment: median 6.0 months/cycles; measured with:** **μmol/L)** | | | | | | | | | | | | |
| 3 | RCT | Serious^1^ | No serious inconsistency | No serious indirectness | Very serious^3^ | Undetected^4^ | 145 | 144 | MD -0.56 (-1.64 to 0.52) | (-13.71, 12.60) | VERY LOW | IMPORTANT |
| **Oestradiol (course of treatment: median 3.0 months/cycles; measured with:** **pg/mL)** | | | | | | | | | | | | |
| 15 | RCT | Serious^1^ | Very serious^2^ | No serious indirectness | No serious imprecision | Undetected | 642 | 639 | MD 2.94 (-1.05, 6.93) | (-13.38, 19.26) | VERY LOW | IMPORTANT |

*(Continued Table 2)*

| **Quality assessment** | | | | | | | **No of patients** | | **Effect estimate** | | **Quality** | **Importance** |
| --- | --- | --- | --- | --- | --- | --- | --- | --- | --- | --- | --- | --- |
| **No of studies** | **Design** | **Risk of bias** | **Inconsistency** | **Indirectness** | **Imprecision** | **Publication bias** | **Experimental** | **Control** | **Point estimate**  **(95% CI)** | **95% Prediction interval** |  |  |
| **Fasting insulin: headache (course of treatment: median 3.0 months/cycles; measured with:** **mIU/L)** | | | | | | | | | | | | |
| 4 | RCT | Serious^1^ | Very serious^2^ | No serious indirectness | No serious imprecision | Undetected^4^ | 175 | 173 | MD -2.48 (-3.85, -1.12) | (-8.05, 3.08) | VERY LOW | IMPORTANT |
| **Body mass index: insomnia (course of treatment: median 4.5 months/cycles; measured with:** **kg/m^2^)** | | | | | | | | | | | | |
| 10 | RCT | Serious^1^ | Very serious^2^ | No serious indirectness | No serious imprecision | Undetected | 373 | 369 | MD -1.78 (-2.53, -1.03) | (-4.25, 0.69) | VERY LOW | IMPORTANT |

Abbreviations: CI = confidence interval, RR = risk ratio, MD = weighted mean difference

^1^ The level of evidence was downgraded by one level if more than 50% of the studies had a moderate or high risk of bias or by two levels if the sensitivity analysis excluding studies with a high risk of bias showed a change in the effect direction.

^2^ The level of evidence was downgraded by one level if the prediction intervals had a partial change compared with the confidence intervals or by two levels if the prediction intervals had a major change compared with the confidence intervals.

^3^ The level of evidence was downgraded by one level if the confidence intervals extended into the no effect line but not beyond the opposite side of the equivalence range or by two levels if the confidence intervals were beyond the opposite side of the equivalence range.

^4^ Tests for detecting publication bias were unavailable because of insufficient data, but the level of evidence was not downgraded.

^5^ Both Egger’s regression and forest plot indicated a significant asymmetry among the effect estimates of individual trials.

The range of equivalence assumed in the assessments of imprecision and inconsistency was an RR of 0.8 to 1.25 for the pregnancy rate, ovulation rate, and miscarriage rate and was an MD of ±0.5 cm^3^ for ovarian volume, ±1 mIU/ml for luteinizing hormone, ±0.5 for follicle-stimulating hormone mIU/ml, ±0.3 for luteinizing hormone to follicle-stimulating hormone ratio, ±5 ng/dl for total testosterone, ±10 pg/ml for oestradiol, ±0.5 μmol/l for dehydroepiandrosterone sulfate, ±1 mIU/l for fasting insulin, and ±0.5 kg/m^2^ for body mass index.
